# Improved Sectional Image Analysis Technique for Evaluating Fiber Orientations in Fiber-Reinforced Cement-Based Materials

**DOI:** 10.3390/ma9010042

**Published:** 2016-01-12

**Authors:** Bang Yeon Lee, Su-Tae Kang, Hae-Bum Yun, Yun Yong Kim

**Affiliations:** 1 School of Architecture, Chonnam National University, 77 Yongbong-ro, Buk-gu, Gwangju 61186, Korea; bylee@jnu.ac.kr; 2 Department of Civil Engineering, Daegu University, 201 Daegudae-ro, Jillyang, Gyeongsan, Gyeongbuk 38453, Korea; stkang@daegu.ac.kr; 3 Department of Civil, Environmental, and Construction Engineering, University of Central Florida, Orlando, FL 32816, USA; Hae-Bum.Yun@ucf.edu; 4 Department of Civil Engineering, Chungnam National University, 99 Daehak-ro, Yuseong-gu, Daejeon 34134, Korea

**Keywords:** fiber, fiber-reinforced concrete, image analysis, orientation

## Abstract

The distribution of fiber orientation is an important factor in determining the mechanical properties of fiber-reinforced concrete. This study proposes a new image analysis technique for improving the evaluation accuracy of fiber orientation distribution in the sectional image of fiber-reinforced concrete. A series of tests on the accuracy of fiber detection and the estimation performance of fiber orientation was performed on artificial fiber images to assess the validity of the proposed technique. The validation test results showed that the proposed technique estimates the distribution of fiber orientation more accurately than the direct measurement of fiber orientation by image analysis.

## 1. Introduction

The first major investigation was made to evaluate the potential of steel fibers as a reinforcement for concrete in the United States during the early 1960s [1]. Since then, a substantial amount of research, development, experimentation and industrial application of steel or synthetic fiber-reinforced concrete has been carried out [2]. The major role of adding fiber is to bridge microcracks and, thus, to improve tensile resistance [3]. Thus, the distribution of fibers strongly influences the resulting mechanical performance of the composite [4,5,6,7,8,9,10]. Short fibers with lengths of 6 to 40 mm, which are randomly distributed in all directions, so as to have isotropic behavior, are commonly used in fiber-reinforced concrete. However, the real fiber distribution is strongly influenced by various factors, such as the fiber characteristics, including the diameter, length and volume fraction, the rheological properties of the matrix, the placing method, the shape of the form, *etc.* Non-uniform fiber distribution decreases the effect of fibers on strengthening the matrix [11,12]. Therefore, it is not reasonable to estimate the uniaxial tensile strength or flexural strength of fiber-reinforced concrete from the assumption of uniformly two-dimensional or three-dimensional distributed fibers. Furthermore, the number of fibers in the sectional image in fiber-reinforced concrete under three-dimensional distribution and two-dimensional distribution are 1/2 and 2/π, respectively, of that under one-dimensional distribution [13]. A directional efficiency coefficient was adopted to consider the effect of fiber orientation distribution on the tensile behavior of fiber-reinforced concrete [14,15,16].

Micromechanically, the fiber orientation, which is the angle of the fiber inclined to the crack plane, influences the fiber pullout load and fiber strength in the matrix. The fiber pullout load increases when the fiber orientation is increased due to the increase of the normal force between the fiber and the matrix, which increases the frictional bond between the fiber and the matrix. This phenomenon is known as the snubbing effect, and currently, an empirical equation between the pullout load of inclined fiber and the pullout load of fiber without an inclination angle is being adopted [17,18]. On the other hand, the fiber strength in a matrix decreases when the fiber orientation is increased due to an additional stress at the exit point of the crack plane by bending [19]. The effect of fiber orientation on the multiple fibers in the composite is taken into consideration in the form of a probability density function for fiber orientation and single fiber pullout load [20].

Several techniques, including image analysis [21,22,23,24,25,26], transmission X-ray imaging [27,28,29,30,31] and alternating current impedance spectroscopy (AC-IS) [32,33], are available for evaluating the fiber dispersion and orientation in a composite made of a cement-based matrix and steel, carbon, glass or synthetic fibers [34]. Among the various techniques, image analysis provides direct information on fiber dispersion and orientation. However, previous studies reported that a two-dimensional image analysis technique may induce a significant systematic error in orientation measurements according to image resolution [21,35]. Therefore, we present a new image analysis technique to improve the evaluation accuracy of fiber orientation distribution in the sectional image of fiber-reinforced cement-based material. The proposed image analysis technique estimates the distribution of fiber orientation from the number of fibers in the sectional images because the number of fibers is dependent on the distribution of the fiber orientation.

## 2. Fiber Distribution Evaluation Method

### 2.1. Image Analysis for the Evaluation of the Fiber Orientation Distribution 

**Figure 1:**
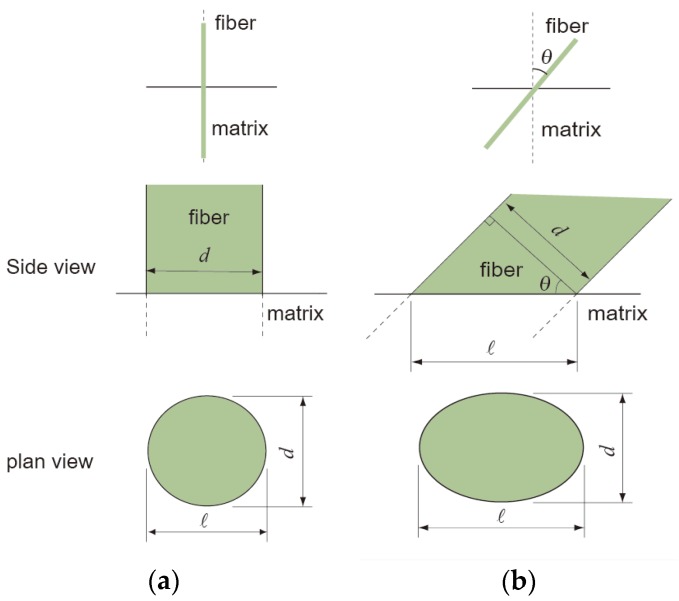
Dimensions of fibers in the side view and plan view on the cutting plane, which is the horizontal line in side view: (**a**) 0° orientation and (**b**) θ orientation.

The distribution characteristics of fiber can be quantitatively evaluated by calculating the coefficient based on the coordinates of the fibers and the shape of the fibers in the cutting plane. To detect the fiber in the fiber images, the color image is converted to a grayscale image. The grayscale image is then converted to a binary image based on a set threshold object detection method, which in turn is based on a thresholding algorithm [36]. In this process, other parts aside from the fibers can be detected as fibers due to having similar brightness to fibers. These misdetected objects are classified on the basis of the threshold of the object’s area, which is determined by the minimum area of randomly-selected fibers. Misdetected objects with a smaller area than the threshold area are deleted. In addition, aggregate fiber images (otherwise known as misdetected fiber images) can be correctly detected by means of the watershed segmentation algorithm and morphological reconstruction [8,37,38]. Over-segmentation by the watershed segmentation algorithm can be minimized by applying a morphological reconstruction [8]. Fiber orientation was defined as the angle between the fiber axis and the normal direction of cutting plane. This is simply calculated by Equation (1) [13].

(1) $$\theta =\text{arccos}\left(\frac{d}{l}\right)=\text{arccos}\left(\frac{d}{d/\cos\theta }\right)$$

where θ, d and l are the inclined angle of the fiber (out-of-plane fiber orientation), the diameter of the fiber and the major axis length of the fiber image, respectively. (Figure 1) The major axis length and diameter, which is the same as the minor axis length, of the fibers were measured by specifying the length (in pixels) of the major axis and minor axis lengths of the ellipse that had the same normalized second central moments as the region.

### 2.2. Effect of the Number of Pixels on the Detection Accuracy

**Figure 2:**
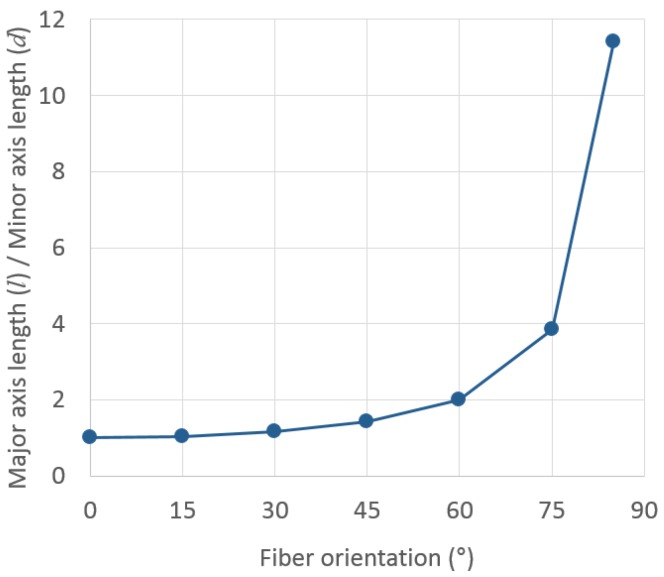
*l*/*d* ratio of the fiber image plotted as a function of fiber orientation, θ.

Table 1 shows artificial fiber images in the cutting plane according to the fiber orientation, and Figure 2 shows the ratio of the major axis length (*l*) to the minor axis length (*d*) of the fiber image with the fiber orientation. As seen in Figure 2, it seems that *l*/*d* is constant below a fiber orientation (θ) of 30°. The difference in *l*/*d* between the fiber images with θ of 0° and 30° is 15.3%. This indicates that an estimation error is unavoidable when we calculate θ based on the *l*/*d* of a fiber image with an actual θ smaller than 30°. In contrast, the difference in *l*/*d* between the fiber images with θ of 0° and 45° is 41.2%; furthermore, the *l*/*d* of a fiber image with θ of 85° is 8.08-times larger than that of a fiber with θ of 45°. This simple numerical simulation shows that the sensitivity of *l*/*d* should sharply increase with a decreasing fiber orientation. This is because *l*/*d* theoretically equals the inverse of cosθ.

**Table 1:** Artificial fiber images in the cutting plane (diameter of 150 pixels).

Fiber Orientation (°)	Fiber Image
0	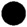
15	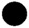
30	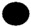
45	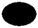
60	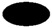
75	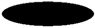
85	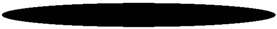

**Figure 3:**
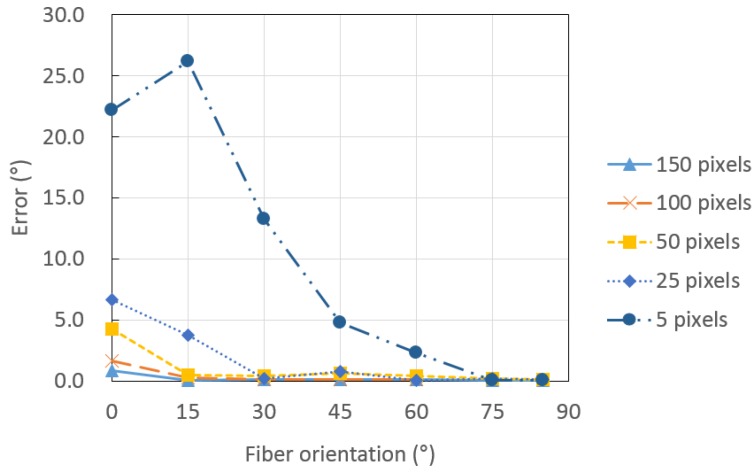
Error of the measured orientation of an artificial fiber image according to the number of pixels in the diameter.

Table 2 lists the fiber orientation measured using an image analysis in an artificial fiber image with a certain orientation according to the number of pixels in the diameter of the fiber. A high number of pixels indicates a high resolution. Figure 3 shows the error of the measured orientation of an artificial fiber image according to the number of pixels in the diameter. The error increases with a decreasing θ and the number of pixels in the diameter of the fiber. This is attributed to the increase in sensitivity with a decreasing θ and the increase of detection error with a decreasing number of pixels in the diameter of the fiber, *i.e.*, a decreasing resolution. The diameter of the synthetic fibers used in a high ductile fiber-reinforced cementitious composite ranges from 10 to 40 μm, and the diameter of steel fibers used in ultra-high performance concrete is about 200 μm [39,40,41]. Table 3 gives the unit pixel length according to the number of pixels and the real diameter of the fiber for three types of fibers with different size diameters. The unit pixel length increases with a decreasing number of pixels in the diameter of the fiber and an increasing real fiber size. If there are five pixels, they represent 200 μm in diameter of steel fiber, and the unit pixel represents 40 μm, which means that one pixel image falsely detected during acquisition or processing may induce an error of 40 μm in measuring the fiber diameter. Therefore, a high enough resolution in relation to the size of the fiber should be employed to prevent false detection. However, a higher resolution requires a larger processing time and memory.

**Table 2:** Measured orientation of the artificial fiber image according to the number of pixels in the diameter.

Number of Pixels in the Diameter of the Fiber	Fiber Orientation (°)						
	0	15	30	45	60	75	85
150	0.8	14.9	29.9	44.9	59.9	74.9	85.0
100	1.7	14.7	29.9	44.9	59.9	74.9	85.0
50	4.3	14.5	29.6	44.4	59.6	74.8	84.9
25	6.6	18.7	30.2	45.7	59.9	74.8	84.9
5	22.2	41.2	43.2	49.8	62.3	75.1	84.9

**Table 3:** Unit pixel length with the number of pixels and the real diameter of the fiber.

Number of Pixels in the Diameter of the Fiber	Real Diameter of the Fiber (μm)		
	10	40	200
150	0.07	0.27	1.33
100	0.10	0.40	2.00
50	0.20	0.80	4.00
25	0.40	1.60	8.00
5	2.00	8.00	40.00

## 3. Image Analysis for Enhancing the Evaluation Accuracy of the Fiber Orientation Distribution

The number of fibers in the sectional images is dependent on the distribution of the fiber orientation. This study suggests that the distribution of the fiber orientation is derived from the number of fibers inversely when enough resolution to ensure the accuracy of fiber detection can be obtained. In this study, a two-parameter exponential function proposed by Xia *et al.* [42] was adopted to express the distribution of fiber orientation, given as follows:

(2) $$g\left(\theta \right)=\frac{\sin\theta^{2p-1}\cos\theta^{2q-1}}{\int_{\theta_{\text{min}}}^{\theta_{\text{max}}}\sin\theta^{2p-1}\cos\theta^{2q-1}d\theta }$$

where *p* and *q* are the shape parameters, which can be used to determine the shape of the probability density function. The parameters *p* and *q* should be more than 0.5, and θ is in a range from 0 to π/2. g(θ) with *p* of one and *q* of 0.5 is the same as sinθ, which is the probabilistic density function for a perfect three-dimensional distribution of fiber orientation. When *p* and *q* are one and one, respectively, g(θ) is 1/π, which is the probabilistic density function for perfect two-dimensional distribution of the fiber orientation. In this study, the parameters *p* and *q* are determined by applying an optimization technique for minimizing the error between the number of fibers measured by image analysis, which is described in Section 2.1, and the number of fibers calculated theoretically. The theoretical number of fibers $N_{f,t}$ is calculated from the volume and diameter of the fiber, the area of the sectional image and the assumed fiber distribution, given as follows:

(3) $$N_{f,t}=\frac{4V_{f}A_{s}}{\pi d^{2}}\int_{\theta_{\text{min}}}^{\theta_{\text{max}}}g\left(\theta \right)\cos\theta d\theta$$

where $V_{f}$ and $A_{s}$ are the fiber content in terms of the volume fraction and the area of the section of fiber-reinforced concrete, respectively.

The optimum technique of the proposed method adopts a direct search, since it does not require any information about the gradient of the objective function and is easy to implement. In this study, a real-valued genetic algorithm is applied to genetic operations for finding the optimal values of *p* and *q* and then estimating the distribution of the fiber orientation. The genetic algorithm is initiated with a set of solutions called populations. Solutions from one population are used to form a new population. This procedure is motivated by the expectation that the new population will be better than the old one. Solutions that are selected to form new solutions are chosen according to their fitness; the more suitable they are, the more opportunities they have to reproduce. This is done by three major processes: selection, crossover and mutation [43,44,45].

The selection is a process in which the best-fit solutions in the population are fit enough to survive and possibly reproduce new solutions for the next generation. The crossover process creates new solutions by combining pairs of old solutions in the current population. This enables the algorithm to extract the best new solutions from different individuals and recombine them into potentially superior new solutions. The mutation process creates new solutions by randomly changing individual old solutions. This prevents all solutions in the population from falling into a local optimum of solved problems. In this study, the population size was set to 200. In order to create a new generation, the roulette wheel selection, the combination of genes with randomly-selected genes from parents’ genes and the addition of a random number taken from a Gaussian distribution with mean zero were adopted for the selection, crossover and mutation process, respectively. When there is no improvement in the objective function for a sequence of consecutive generations of length 50, the process is stopped.

The fitness function is expressed in the form of Equation (4).

(4) $$f_{\text{fitness}}\left(p,q\right)=\left|N_{f,t}\left(p,q\right)-N_{f,m}\right|$$

where $N_{f,m}$ is the measured number of fibers by image analysis.

## 4. Validation of the Proposed Technique

**Figure 4:**
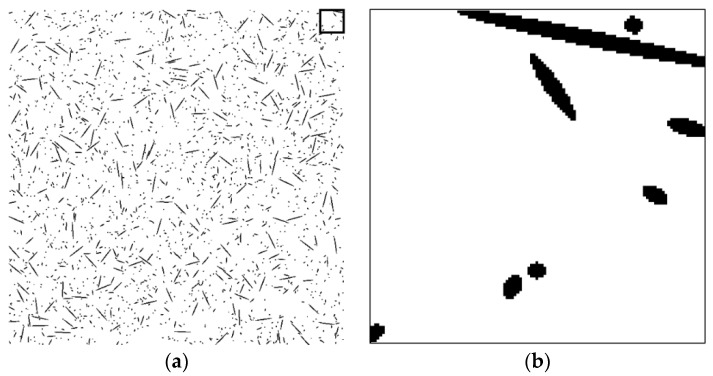
Artificial fiber images with a diameter of five pixels in a three-dimensional random distribution in the area of 2000 pixels by 2000 pixels (I05-3): (**a**) section image and (**b**) magnified image.

**Figure 5:**
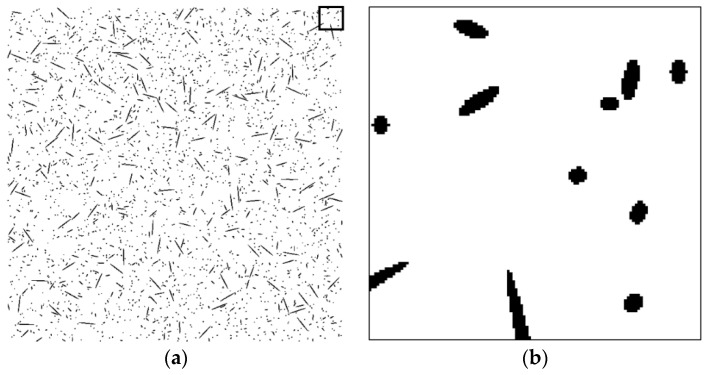
Artificial fiber images with a diameter of five pixels in a two-dimensional random distribution in the area of 2000 pixels by 2000 pixels (I05-2): (**a**) section image and (**b**) magnified image.

**Figure 6:**
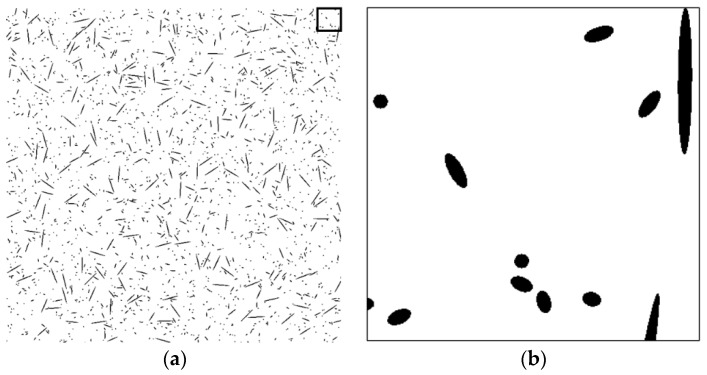
Artificial fiber images with a diameter of 15 pixels in a three-dimensional random distribution in the area of 6000 pixels by 6000 pixels (I15-3): (**a**) section image and (**b**) magnified image.

**Figure 7:**
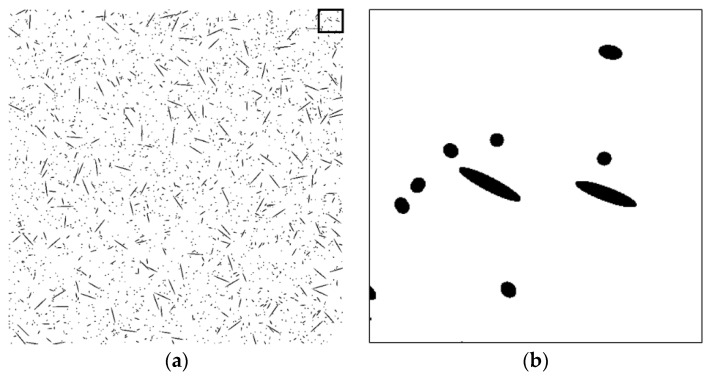
Artificial fiber images with a diameter of 15 pixels in a two-dimensional random distribution in the area of 6000 pixels by 6000 pixels (I15-2): (**a**) section image and (**b**) magnified image.

**Figure 8:**
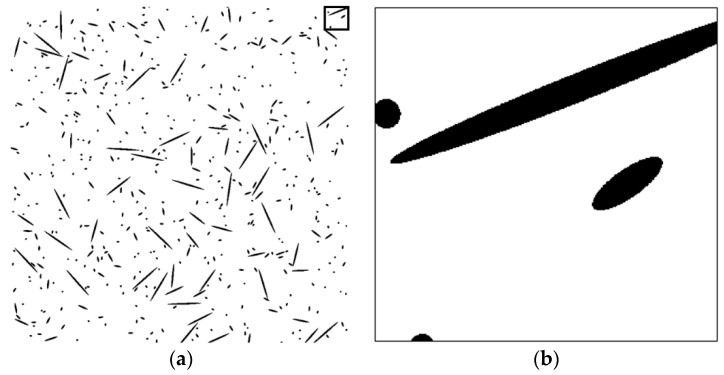
Artificial fiber images with a diameter of 25 pixels in a three-dimensional random distribution in the area of 5000 pixels by 5000 pixels (I25-3): (**a**) section image and (**b**) magnified image.

**Figure 9:**
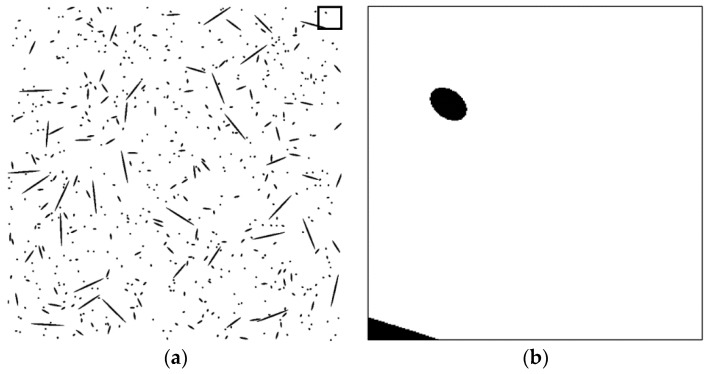
Artificial fiber images with a diameter of 25 pixels in a two-dimensional random distribution in the area of 5000 pixels by 5000 pixels (I25-2): (**a**) section image and (**b**) magnified image.

To assess the validity of the proposed technique, a series of tests was performed on artificial fiber images. Artificial section fiber images with three sizes of fibers and two- and three-dimensional random distributions of fiber orientation were tested (Table 4). The total fiber volume was assumed to be 2.0 vol%. The number of fibers was calculated from the fiber volume fraction, the area of the images, the diameter of the fibers and the distribution characteristics of the fiber orientation. Figure 4, Figure 5, Figure 6, Figure 7, Figure 8 and Figure 9 show the sectional fiber images made artificially for the test of the validation. As shown in Figure 4b and Figure 5b, with the diameter of five pixels representing the diameter of the fiber, the surface of the fiber image is not a smooth curve, but a series of discontinuous lines. On the other hand, the surfaces of the fiber images are smoother with an increasing resolution and the number of pixels representing the diameter of the fiber from five to twenty five.

**Table 4:** Images to assess the performance of the proposed technique.

Image ID	Number of Pixels in the Diameter of the Fiber	Area (Pixel^2^)	Dimension of the Fiber Orientation Distribution	Number of Fibers
I05-3	5	2000 × 2000	3	2038
I05-2			2	2594
I15-3	15	6000 × 6000	3	2038
I15-2			2	2594
I25-3	25	5000 × 5000	3	510
I25-2			2	649

**Figure 10:**
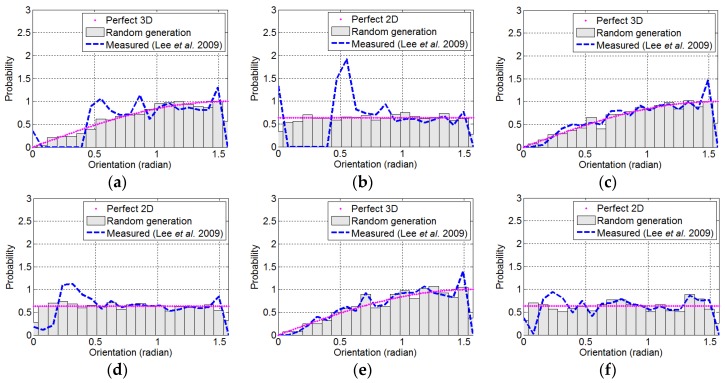
Probability density functions of the orientation of randomly-generated artificial fibers and the orientation measured by the image analysis technique proposed by Lee *et al.* (2009): (**a**) I05-3; (**b**) I05-2; (**c**) I15-3; (**d**) I15-2; (**e**) I25-3; (**f**) I25-2.

**Figure 11:**
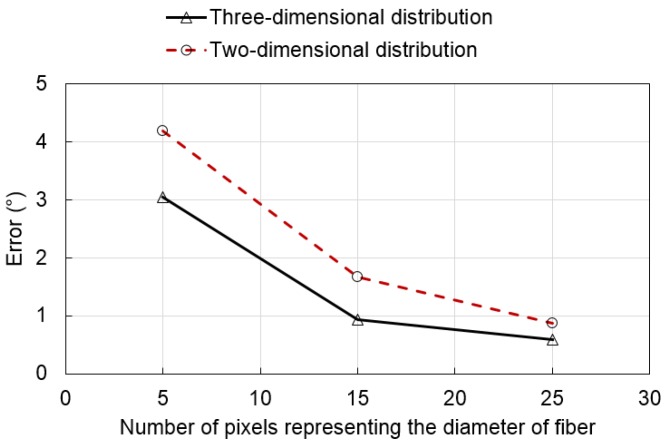
Average error per fiber of the measured orientation of the artificial fiber images according to the number of pixels in the diameter.

Figure 10 shows the probability density functions of the fiber orientation according to the number of pixels representing the diameter of the fiber and the distribution characteristics (two- or three-dimensional distribution). The fiber orientation was measured using the image processing technique described in Section 2.1 based on Lee’s technique [8]. The randomly-generated artificial fibers show probability density functions similar to those of perfectly uniform two- or three-dimensional distributions. However, the probability density functions from the direct measurement of fiber orientation by image analysis are considerably different from those of real distributions, especially in the region of low θ. The error of the two-dimensional distribution is larger than that of the three-dimensional distribution. This is attributed to the fact that two-dimensional distribution images have a higher probability for low θ fibers than that of three-dimensional distribution images. The error decreased with an increasing number of pixels, which represents the diameter of the fiber. This can be expected from the investigation in Section 2.2. Figure 11 shows the average errors per fiber of the measured orientation of the artificial fiber images from Figure 4, Figure 5, Figure 6, Figure 7, Figure 8 and Figure 9 according to the number of pixels representing the diameter of the fibers. The average error per fiber in the three-dimensional distribution was 32.4% lower than that in the two-dimensional distribution. In contrast, the average error per fiber in the two-dimensional distribution more sharply decreased with an increasing number of pixels compared to that in the three-dimensional distribution. Average errors per fiber of the I15-2 and I25-2 images decreased by 70.0% and 80.8% compared to I05-2, respectively, while average errors per fiber of the I15-3 and I25-3 images decreased by 60.1% and 79.3% compared to I05-3, respectively. This is also due to the higher proportion of low θ fibers in a two-dimensional distribution image compared to that of a three-dimensional distribution image.

**Figure 12:**
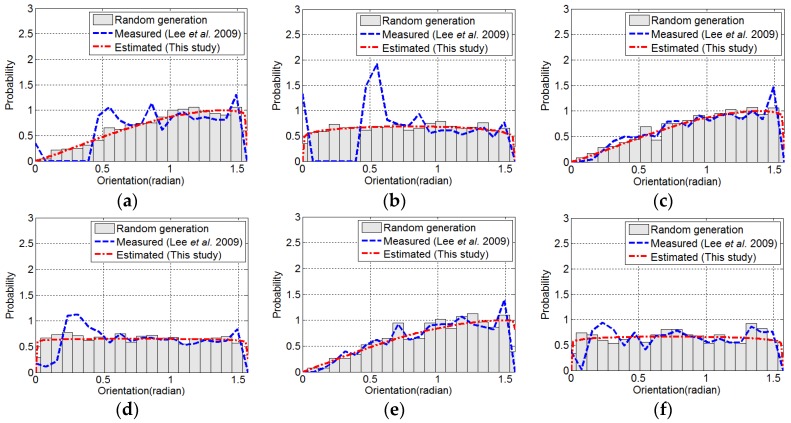
Comparison between the measured distribution and the estimated distribution by the technique proposed in this study: (**a**) I05-3; (**b**) I05-2; (**c**) I15-3; (**d**) I15-2; (**e**) I25-3; (**f**) I25-2.

**Figure 13:**
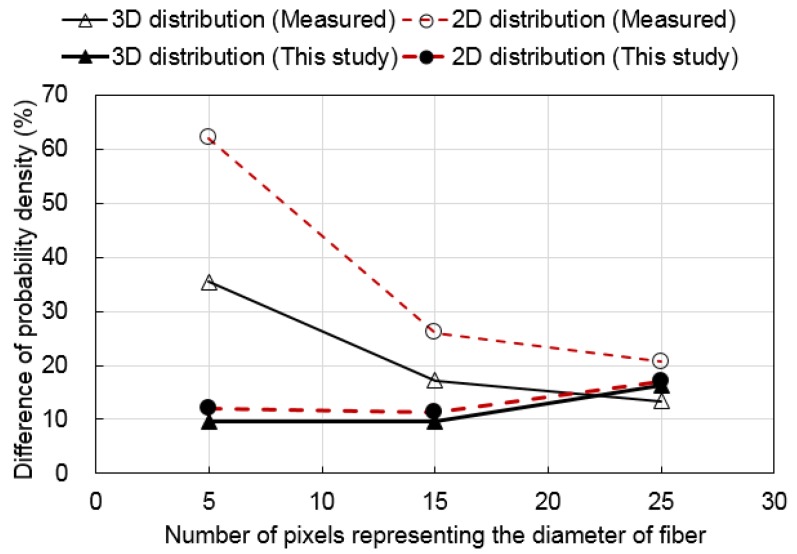
Comparison between the measured distribution and the estimated distribution by the technique proposed in this study.

Figure 12 shows the probability density functions of the real orientation of the fibers, which was randomly generated artificially, the direct measurement of the fiber orientation by the image analysis proposed by Lee *et al.* [8] and the estimated probability density functions from the technique proposed in this study. Figure 13 shows the difference in the probability densities from the direct measurement of the fiber orientation by image analysis and the estimation by the technique proposed in this study compared to that of the real orientation. No difference indicates that the fiber orientations of all of the fibers are exactly measured or estimated with the real fiber orientation by the techniques. With the three-dimensional distribution, the differences in the probability density for the I05-03 and I15-03 images decreased by 72.7% and 43.7%, respectively, by the proposed technique compared to the previous technique (direct measurement). However, the differences in the probability density for the I25-03 image was increased by 21.9% with the proposed technique compared to the previous technique. This may be attributed to the larger variation of the probability densities of the fiber orientation of the I25-03 image compared to those of I05-03 and I15-03. With the two-dimensional distribution, the differences in the probability density for the I05-02, I15-02 and I25-02 images decreased by 80.6%, 56.1% and 17.1%, respectively, with the proposed technique compared to the previous technique. The differences in the probability density converged with an increasing number of pixels representing the diameter of the fiber regardless of the dimensions of the distribution of the fiber orientation. The test results confirmed that the technique proposed in this study provided better estimation performance than the previous technique, especially when there were two-dimensional distributions and a small number of pixels representing the diameter of the fiber. The image analysis technique proposed in this study can be used to assess or analyze with more accuracy the effects of the fiber orientation on the mechanical properties of fiber-reinforced concrete when the number of pixels representing the diameter of the fiber is limited in the process of image acquisition.

## 5. Conclusions

We proposed a new image analysis technique to estimate the distribution of fiber orientation in a sectional image of fiber-reinforced concrete. A series of experimental and analytical investigations with artificial fiber images was carried out to assess the validity of this technique. The following conclusions can be drawn from the results:

(1) We investigated the effect of the number of pixels representing the diameter of the fiber and fiber orientation on the detection accuracy. The error increased with a decreasing fiber orientation and the number of pixels in the diameter of the fiber. We attributed this to an increase in sensitivity with a decreasing fiber orientation and an increase in the detection error with a decreasing number of pixels in the diameter of the fiber, *i.e.*, the decreasing resolution.

(2) The proposed technique estimates the distribution of fiber orientation by finding optimal distribution functions matching the measured number of fibers by an image analysis with the theoretical number of fibers calculated from the volume and diameter of the fiber, the area of the sectional image and the assumed fiber distribution.

(3) Validation tests using artificial fiber images according to the size of the fiber images and the dimensions of the fiber orientation confirmed that the technique proposed in this study ensures better evaluation performance than that by direct measurement of the fiber orientation from image analysis, especially when there is a two-dimensional distribution and a small number of pixels representing the diameter of the fiber.
